# Black TiO_x_ Films with Photothermal-Assisted Photocatalytic Activity Prepared by Reactive Sputtering

**DOI:** 10.3390/ma14102508

**Published:** 2021-05-12

**Authors:** Quan Mao, Meng Liu, Yajie Li, Yuquan Wei, Yong Yang, Zhengren Huang

**Affiliations:** 1 State Key Laboratory of High Performance Ceramics and Superfine Microstructures, Shanghai Institute of Ceramics, Chinese Academy of Sciences, 1295 Dingxi Road, Shanghai 200050, China; maoquan@mail.ustc.edu.cn (Q.M.); liumeng1@shanghaitech.edu.cn (M.L.); liyj@shanghaitech.edu.cn (Y.L.); weiyuquan@mail.sic.ac.cn (Y.W.); 2 Center of Materials Science and Optoelectronics Engineering, University of Chinese Academy of Sciences, Beijing 100049, China; 3 School of Physical Science and Technology, ShanghaiTech University, Shanghai 201210, China

**Keywords:** black TiO_x_, reactive sputtering, photothermal-assisted photocatalysis

## Abstract

Titanium oxide is widely applied as a photocatalyst. However, its low efficiency and narrow light absorption range are two main disadvantages that severely impede its practical application. In this work, black TiO_x_ films with different chemical compositions were fabricated by tuning target voltage and controlling O_2_ flow during reactive DC magnetron sputtering. The optimized TiO_x_ films with mixed phases (TiO, Ti_2_O_3_, Ti_3_O_5_, and TiO_2_) exhibited fantastic photothermal and photocatalytic activity by combining high light-absorptive Ti_2_O_3_ and Ti_3_O_5_ phases with the photocatalytic TiO_2_ phase. The sample prepared with oxygen flow at 5.6 ± 0.2 sccm and target voltage near 400 V exhibited excellent optical absorbance of 89.29% under visible light, which could improve surface temperature to 114 °C under sunlight. This film could degrade Rhodamine-B up to 74% after 150 min of UV irradiation. In a word, this work provides a guideline for fabricating black TiO_x_ films with photothermal-assisted photocatalytic activity by reactive DC magnetron sputtering, which could avoid the usage of hydrogen and is convenient for quantity preparation.

## 1. Introduction

TiO_2_ is a commercial photocatalyst that has been widely applied in antibacterial disinfection, water treatment, and air purification due to its nontoxic, harmless, and chemistry-stable features [1]. However, its wide bandgap hinders its optical absorption range to ultraviolet light, which occupies less than 5% of the whole solar energy [2]. Therefore, it is necessary to broaden its optical absorbance range for practical application.

In 2011, Chen et al. [3] reported a novel kind of “black TiO_x_” nanoparticles with an optical absorption starting from 1.0 eV (~1200 nm) by a hydrogenated method, which made significant improvements in visible-light photocatalytic activity on water splitting and dye degradation. Since then, a large number of methods [4] have been used to obtain black TiO_x_ nanoparticles, such as hydrogen thermal treatment [5], hydrogen plasma [6], electrochemical reduction, and chemical reduction. Moreover, the photothermal-assisted photocatalyst system with a light absorber/semiconductor composite (LASC) has been demonstrated beneficial for the photocatalytic reaction [7,8,9]. LASC systems composed of black TiO_x_ with high absorbance might bring substantial photothermal conversion efficiency for photocatalytic application.

In general, black TiO_x_ film exhibits more catalytic application potential than TiO_x_ powders [10]. Therefore, all kinds of methods such as electrospinning [11], the sol–gel process [12], pulsed laser deposition (PLD) [13], hydrogen treating [14], H_2_ plasma-assisted deposition [6], and reactive magnetron sputtering [15] have been used to fabricate black TiO_x_ films. Among those methods, reactive magnetron sputtering is widely applied to fabricate films for industrial production, employing gas to react with sputtered atoms to obtain compounds. This method can easily adjust the composition and structure of film with high quality, adhesion, and uniformity, avoiding the safety problem caused by hydrogen.

The structure and composition of black TiO_x_ films can be tuned by controlling the O_2_/Ar flow ratio in reactive magnetron sputtering to increase its optical absorbance of visible light and photocatalytic activity. Zapata-Torres et al. [15] studied the oxygen mass flow during RF reactive magnetron sputtering to improve the infrared photocatalytic activity of black TiO_x_ films. Adriano et al. [16] also obtained TiO_x_ films with tunable crystal structure by fine control of the O_2_/Ar ratio during reactive DC magnetron sputtering. However, based on these studies [17,18], a surplus oxygen flow would oxidize the Ti target and result in continuous target poisoning, which may influence film composition and the sputtering speed. The changes in gas partial pressure can be fast and directly reflected by the cathode voltage during reactive magnetron sputtering [18,19], which helps to stabilize sputtering and avoids target poison. Therefore, it is very important to carefully adjust the target voltage in the reactive DC magnetron sputtering process for fabricating black TiOx films.

In this work, black TiO_x_ films were successfully fabricated by reactive DC magnetron sputtering with a Ti target in an oxygen and argon mixed atmosphere. By tuning the target voltage and controlling the oxygen flow ratio, black TiO_x_ films with different compositions could be obtained. We analyzed the phase, surface morphology, optical absorbance, and Ti valence of these films. The photothermal and photocatalytic activities of the films were also measured, which provided a guide for fabricating black TiO_x_ films by reactive DC magnetron sputtering.

## 2. Materials and Methods

### 2.1. Preparation of Black TiO_x_ Films

Black TiO_x_ films were prepared by reactive DC magnetron sputtering (MSP-3200, Beijing Chuangshiweina Technology Co., LTD, Beijing, China) on monocrystalline silicon substrates, FTO glass, and glass slides (2.55 cm × 7.65 cm) at room temperature. The films deposited on monocrystalline silicon substrates were used for morphology, phase, and composition research. The films on FTO glass were used to test the photocurrent, and the films on glass slides were used to test the photothermal effect and photocatalytic activity. To control the chemical compositions in films, we controlled the target voltage and the oxygen flow ratio. The chemical equation during reactive sputtering is shown as follows:

(1) $$Ti+\frac{x}{2}O_{2}\to TiO_{x}$$

where 0 ≤ x ≤ 2. The Ti (99.99% purity) target was a disc with a diameter of 101 mm and a thickness of 6 mm. The distance between the target and substrate was 120 mm, and the angle between the target and the substrate normal was 29°. The rotation speed of the substrate was 20 r/min to ensure the unity of the films. We fixed the total pressure during sputtering at 0.5 Pa. The sputtering power was 150 W with a bias of 100 V. We used mass flow controllers to control argon (99.99% purity) and oxygen (99.99% purity). Table 1 shows the target voltage, gas flow, and corresponding sample numbers.

### 2.2. Materials Characterization

The crystal structures of films were characterized by an X-ray diffractometer (D8 ADVANCE, Copyright Bruker, Billerica, MA, USA) with Cu Kα (λ = 1.5406 Å, 40 Kv, 30 mA) radiation. The surface morphologies and thickness of films were determined by a scanning electron microscope (SU 8220, HITACHI, Tokyo, Japan) at an accelerating voltage of 10 kV. The peak value (PV), the root mean square (RMS) value, and the roughness average (Ra) value of films was gathered by an atomic force microscope (NTEGRA, NT-MDT, Moscow, Russia). We used the tapping mode with 256 × 256 points in an area of 2 um × 2 um. The AFM data estimated the average particle size. The “grain analyze” function in the “IA trunk” was used to count the grain amount in the areas. The element composition of films was detected by EDS (equipped on the SEM), and the valence of elements was tested by an X-ray photoelectron spectrometer (ESCAlab250, Thermo Fisher Scientific, Waltham, MA, USA) with Al Kα radiation. The surface of films and after 10 s of 20 kV Ar ion etching was used to study the general survey and the binding energy of O 1s, Ti 2p, and C 1s. The binding energy of C 1s at 285 eV was used as a reference to calibrate the peak positions. The XPS results dealt with “Shirley” methods to cut background and 80%/20% Gauss/Lorentz peak shape to obtain fitting curves of different binding energies. The valence percentage was calculated by the formula:

*Ti*^*n*+^% = *A*_*n*_/*A*_0_ (2)

where *A_n_* is the peak area of *Ti^n+^*, and *A*_0_ is the total area of Ti 2p binding energy.

The films deposited on glass slides were used to study their optical transmittance and reflectance. The transmittance and reflection spectra were gathered by an ultraviolet-visible spectrophotometer (PerkinElmer Lambda 950, equipped with an integrating ball, Waltham, MA, USA). The absorbance was calculated by the formula:

*A* = 100% − *T* − *R* (3)

where *A* is the absorbance, *T* is the transmittance, and *R* is the reflection.

### 2.3. Evaluation of Photothermal Effect and Photocatalytic Activity

A Xenon lamp (PLS-SXE300/300UV, Perfect Light, 100 mW/cm^2^, Beijing, China) was used to simulate sunlight to measure the photocatalytic activity and photothermal effect under visible light. The samples were placed 30 cm under the light on a foam mat. An infrared thermometer gun (FLUKE, 62 Mini IR THERMOMETER, Everett, WA, USA) was used to test the surface temperature of the films. A filter that could cut light wavelength under 400 nm was used to avoid ultraviolet light. A low-pressure mercury lamp (Philips, 25 W, 8 mW/cm^2^, Amsterdam, the Netherlands) was used as a light source to assess the UV photocatalytic activity of the films. The samples were placed fully into a beaker with 30 mL 10 mg/L Rhodamine-B aqueous solutions and 30 cm under the light source. Before the degradation process, the solution was placed in a dark box for 2 h for adsorption equilibrium, and the solution was stirred by magnetic stirring apparatus. To avoid the loss of solvent, we added a culture dish on the top of the beaker. The evaporation water might flow back to the beaker. The dye concentration in the solution has a linear relation to its absorption [20,21]. The assessment of photocatalyst activity was made by the absorption change of solution at 553 nm after the degradation process under the light. An electrochemical workstation (CHI-600E, Shanghai, China) and a three-electrode system were applied to test photogenerated current. The platinum plate electrode was used as the counter electrode, the film on FTO glass as the working electrode, and the AgCl as the reference electrode in an aqueous 1 mol/L NaCl electrolyte.

## 3. Results and Discussion

### 3.1. Structure, Chemical Component, and Morphology

**Figure 1:**
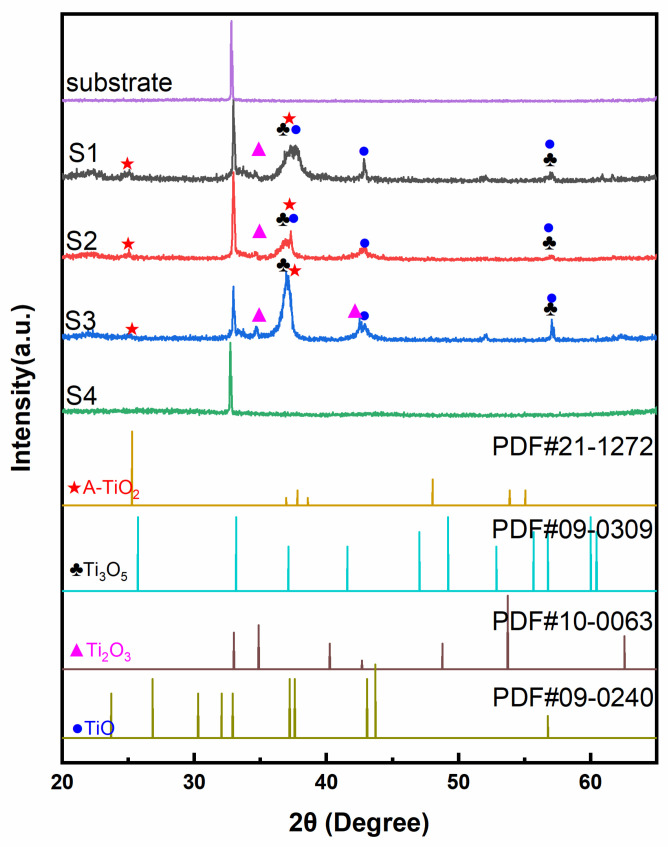
The XRD patterns of Samples S1, S2, S3, and S4, substrate, and pure anatase TiO_2_.

The crystalline structures of as-prepared TiO_x_ films were analyzed by XRD and shown in Figure 1. Due to the inadequate oxygen condition during the sputtering process, it could generate many titanium simply oxides. According to PDF#21-1272, PDF#09-0309, PDF#10-0063, and PDF#09-0240, there were several kinds of titanium oxides in these films, including anatase TiO_2_, Ti_3_O_5_, Ti_2_O_3_, and TiO. As the oxygen flow increased, the main phase changed from Ti to TiO, to Ti_2_O_3_, to Ti_3_O_5_, and TiO_2_. The main crystal plane (101) of anatase TiO_2_ at 2θ ≈ 25.3° was hard to recognize. Anatase TiO_2_ starts to crystalize at 300 °C [22,23]. Thus, this phase was difficult to form while the temperature of the chamber was lower than 80 °C. From Samples S1 to S3, the (110) (2θ ≈ 34.8°) and (202) (2θ ≈ 42.7°) planes of Ti_2_O_3_ became more apparent with the increased oxygen flow ratio. The diffraction peak (2θ ≈ 37.1°) of Sample S3 became sharp and intense for forming the Ti_3_O_5_ (130) plane. Ti_3_O_5_ could crystalize at 120 °C [24], which was easy to form and crystallize during the sputtering process. The TiO (−211) plane (2θ ≈ 37.6°) disappeared in Sample S3 due to the decreased content of the TiO with the increased oxygen flow ratio. Sample S4 was similar to the bare substrate. When the oxygen flow ratio was over 15%, the target was poisoned to form a few amorphous TiO_2_ [17,18]. Proper oxygen flow ratio (Sample S3) was significant to the formation of Ti_2_O_3_ and Ti_3_O_5_.

**Figure 2:**
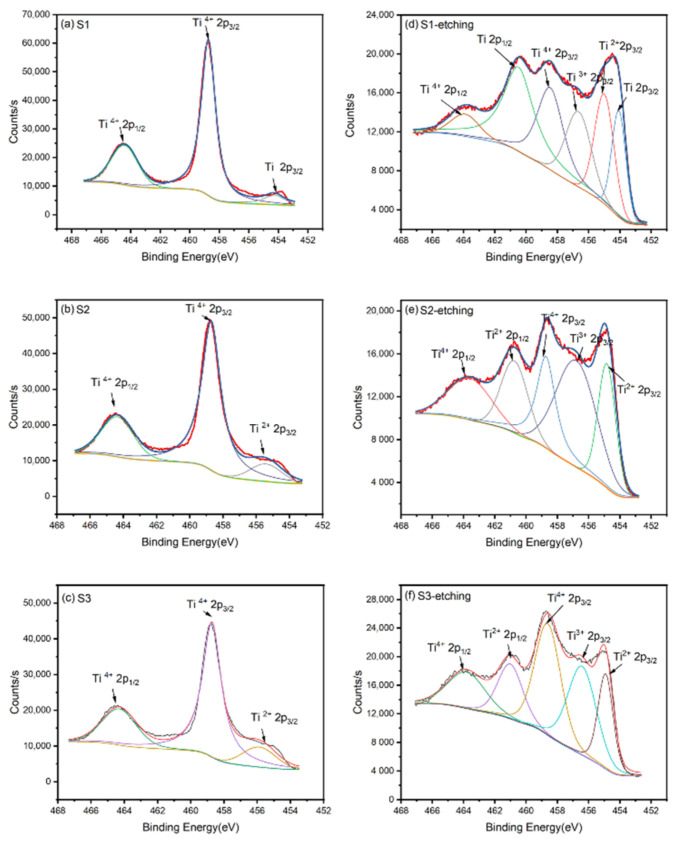
The binding energy of Ti 2p on the surface of the films before etching: (**a**) S1, (**b**) S2, and (**c**) S3; the binding energy of Ti 2p on the surface of the films after etching: (**d**) S1, (**e**) S2, and (**f**) S3.

In order to obtain an accurate composition of these films, XPS was applied to analyze the binding energy of Ti 2p and obtain the composition information of these films. As shown in Figure 2a–c, the binding energies of Ti 2p on the surface before etching were mainly 458.7 and 464.5 eV, corresponding to Ti^4+^ 2p_3/2_(TiO_2_) and Ti^4+^ 2p_1/2_(TiO_2_), respectively. As an active metal, Ti could react with oxygen in the air to form a layer of TiO_2_ on its surface. Thus, although these films were prepared with different oxygen flows, their surfaces were oxidized to TiO_2_, which was the same as the work of Zapata-Torres [15] et al. To obtain the inner information of these films, the TiO_2_ on the surface should be removed. However, it is worth noting that ion etching will reduce Ti^4+^ to Ti^3+^ and Ti^2+^ [25]. Thus, we etched the surfaces of all samples in 10 s to limit the reduction of Ti^4+^. Figure 2d–f indicates the binding energy of Ti 2p on the surface after etching. From the XPS spectra, the peaks of Ti, Ti^2+^, and Ti^3+^ appeared for the inner titanium simply oxides. Thus, as noted in the XRD pattern, the XPS spectra also proved the phase-changing trend of TiO, Ti_2_O_3_, Ti_3_O_5_, and TiO_2_. Zapata-Torres [15] et al. also obtained similar Ti_2_O_3_ and TiO by RF sputtering. Table 2 indicates that the valence state of Ti is rising with the increase in the oxygen flow ratio. The O 1s XPS spectra and related peak data are shown in Figure S1 and Tables S1–S6, which can be attributed to the Ti-O bond from the films and the C-O bond from external organic pollutants.

XPS is sensitive to the surface, which makes it hard to obtain complete information of the films. EDS can detect deeper information, and the O: Ti ratio is shown in Table 2. The fitting curves of EDS spectra are shown in Figures S2–S4, Tables S7–S9. With the increase in the oxygen flow rate, the atomic ratio of oxygen to titanium also increases.

**Figure 3:**
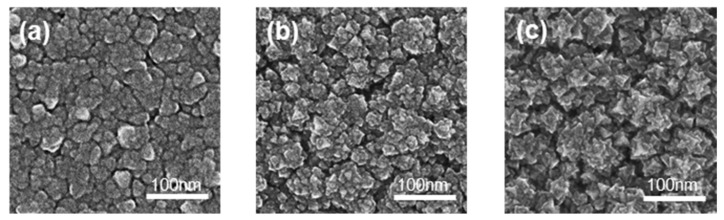
The SEM images of the films: (**a**) S1; (**b**) S2; (**c**) S3.

Morphologies, roughness, average particle size, and thickness of films significantly affect photocatalytic activity. SEM and AFM were applied to detect the above information, which is shown in Figure 3, Figures S5 and S6, and Table 3. The thickness of the films increased with the rise in O_2_ flow and target voltage. Due to the rising voltage, the electric field intensity also increased, leading to increased plasma density for a higher sputtering rate to increase the thickness. Roughness is always proportional to thickness. Sample S1 was relatively flat with many clusters and shallow ravines. The clusters in Sample S2 exhibited more obvious edges and more holes than Sample S1. The clusters in Sample S3 showed the sharpest edges and corners. Combining the XRD patterns with the surface morphologies, Sample S3 exhibited the best crystallinity and roughness.

Good crystallinity is considered to be a favorable factor for the TiO_2_ photocatalytic reaction because of fewer defects for recombination centers [26]. Commonly, when the film has a rough surface and a higher specific surface area, it will produce more reactive sites, which are favorable for the photocatalytic reaction [27]. Increasing the roughness of films could improve photocatalytic activity [28,29]. In the work of Adriano et al. [16], when the O_2_/Ar flow ratio is below 1/7, the roughness rises with the increase in the O_2_/Ar flow ratio, consistent with our results.

### 3.2. Optical Absorbance and Photothermal Conversion

**Figure 4:**
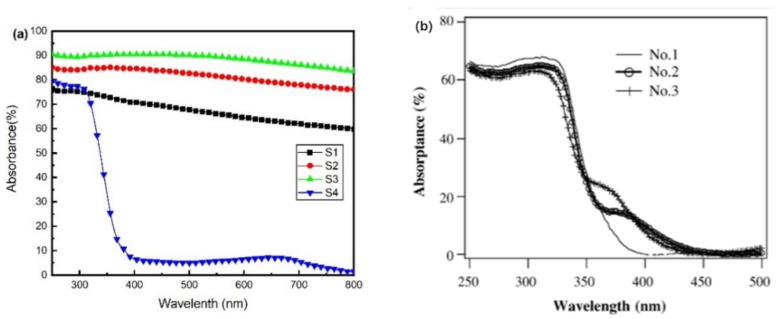
(**a**) The ultraviolet-visible absorbance of Samples S1, S2, S3, and S4; (**b**) the absorbance of films treated by H_2_ or N_2_, No.1: untreated TiO_2_, No.2: TiO_2_ treated by H_2_, No.3: TiO_2_ treated by N_2_.

Figure 4a presents the calculated absorbance spectra of the films. The average optical absorbance of each sample from 250 to 800 nm is shown in Table 1. All films showed high optical absorbance except transparent S4. The average absorbance first increased before the oxygen flow rose to 5.6 sccm and finally decreased at 6 sccm. When the oxygen was abundant, it could produce stoichiometric TiO_2_, leading to a very low absorbance in the visible region. Among these samples, S3 exhibited the highest average absorbance, up to 89.29%. Figure 4b reveals the absorbance spectra of TiO_2_ films treated with H_2_ or N_2_ [30]. Due to the formation of black TiO, Ti_2_O_3_, and Ti_3_O_5_, the films in our work exhibit increased absorbance compared with these treated TiO_2_ films [30]. After three minutes of simulated sunlight irradiation, an apparent temperature rise could be observed. While the room temperature was 29 °C, the temperature of transparent sample S4 rose to 54 °C, and black S1, S2, and S3 increased to 72, 102, and 114 °C, respectively.

Both Ti_2_O_3_ and Ti_3_O_5_ have been reported for their high-performance photothermal conversion [9,31]. The increase in surface temperature shows a significant positive correlation with the content of Ti_2_O_3_ and Ti_3_O_5_ phases in black films. Thus, the reactive sputtering technology, which could introduce Ti_2_O_3_ and Ti_3_O_5_ into the TiO_2_ films, might adjust the light absorption to the visible range. The films containing simply titanium oxides exhibited the potential to be excellent photothermal materials.

### 3.3. Photocatalytic Activity

**Figure 5:**
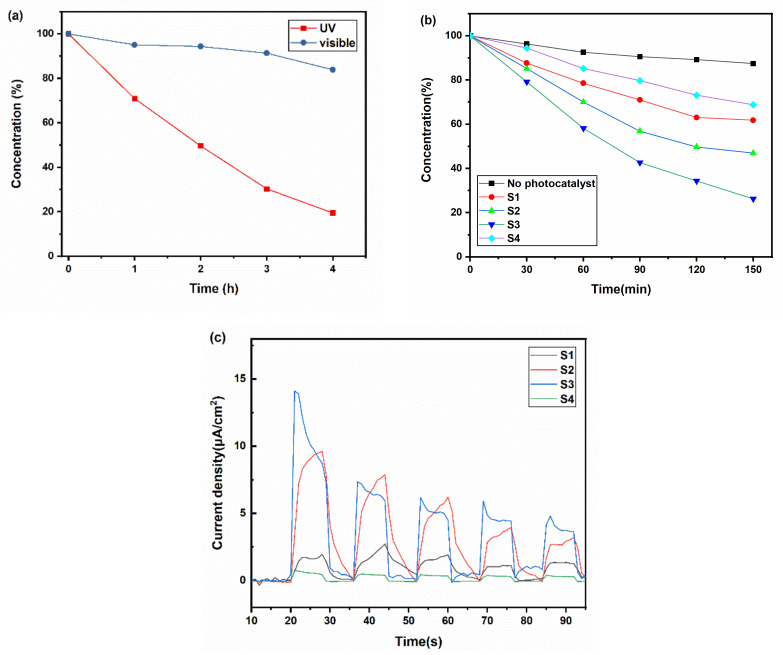
(**a**) The dye degradation dynamic curve of Sample S3 under visible light and ultraviolet; (**b**) the dye degradation dynamic curve of Sample S1, S2, S3, and S4 under UV light; (**c**) the photoelectric current of Sample S1, S2, S3, and S4 under visible light.

To determine the suitable light source for the photocatalytic application of the black TiO_x_ films, we tested their photocatalytic activity under visible light (cutting UV light) and UV light, as shown in Figure 5a. It can be found that the degradation speed of RhB under ultraviolet was faster than that under visible light. Thus, UV light seemed to be the most suitable light source for its application.

Films with different absorbances were tested under UV light to understand how the photothermal effect influenced the photocatalytic process. As shown in Figure 5b, after 150 min, the dye concentration of S1, S2, S3, and S4 decreased to 69%, 62%, 47%, and 26%, respectively. The temperature of the solution contained S1, S2, S3, and S4 was 48, 53, 56, and 40 °C, respectively (S4 was the same as the solution without photocatalysts). The temperature of the solution has a significant influence during the dye degradation process. According to the Arrhenius equation, the heat produced by the photothermal effects can decrease the activation energy and increase the pre-exponential factor, which benefits the degradation of dye. The temperature does a positive influence, identical to the results from Janus et al. [32] The RhB photocatalytic degradation rate in the blackest sample S3 was 2.4 times that of the transparent sample S4. Figure 5c indicates that Sample S3 exhibited a larger photoelectric current than others, which rose to 15 times compared with the transparent sample S4. Samples S1 and S2 also exhibited larger photoelectric currents, which revealed that the mixed phases and good crystallinity could improve the separation effect of photogenerated electron–hole pairs.

Table 4 summarizes the photocatalytic efficiency of several TiO_2_ films by sputtering methods. Compared to these results, the black TiO_x_ films in our work presented better photocatalytic activity than the TiO_2_ films without doping [20,21], even better than the film combined with ZnO [33]. The films in our work can reach the level of the films made by hydrogenate method [34]. However, compared with TiN/TiO_2_ films [35], we still have a gap in visible photocatalytic activity. Different structure designs based on our work may improve the visible-light photocatalytic efficiency in the future.

Recently, many photothermal-assisted photocatalysts have been reported [7,8,36]. Most of them combined high-performance photothermal conversion materials with high-performance photocatalysts to build co-photocatalysts. According to the results of XRD, XPS, and EDS, oxides including TiO, Ti_2_O_3_, Ti_3_O_5_, and TiO_2_ exist in these films. Therefore, these oxides constitute LASC systems that could improve photocatalytic activity.

Among these samples, Sample S3 exhibited the best photothermal and photocatalytic activities. By controlling the oxygen flow at 5.6 ± 0.2 sccm and target voltage near 400 V, a film containing mostly Ti_2_O_3_ and Ti_3_O_5_, with high optical absorbance of 89%, could be fabricated. As the absorbance increased, the photothermal efficiency improved too. Most ultraviolet sources also generate some visible light, which could be absorbed by the Ti_2_O_3_ and Ti_3_O_5_ phases in the films, causing the rise in temperature during the reaction process and benefiting photocatalytic activity. Depending on XRD and SEM results, Sample S3 exhibited the best crystallinity and roughest surface, benefiting the dye degradation. According to the photoelectric currents test, the mixed phases in the film and good crystallinity led to low recombination of the photogenerated electron–hole pair, which increased the lifetime of the photogenerated carrier for better photocatalytic performance.

Unlike many black TiO_2_ materials, the black TiO_x_ films in this work did not show ideal photocatalytic activities in the visible region. Most researchers reported that they hydrogenated the white TiO_2_ to build a disordered surface structure with an ordered white TiO_2_ core and then obtained impressive solar-driven black TiO_2_ photocatalysts [4]. During the hydrogenation process, the surface could generate many oxygen vacancies, producing an intermediate level to narrow the bandgap. However, in our methods, the O: Ti atoms ratio was far below two, and it could form many Ti_2_O_3_ and Ti_3_O_5_, which exhibited good optical absorbance but low photocatalytic activity. The increased photocatalytic efficiency was mainly owing to its photothermal effects.

In a word, we endowed the black TiOx films of photothermal and photocatalytic activity at the same time by combining Ti_2_O_3_, Ti_3_O_5_, and TiO_2_ through reactive sputtering. The photothermal effects of Ti_2_O_3_ and Ti_3_O_5_ and the formation of the LASC system significantly improve the photocatalytic activity in dye degradation.

## 4. Conclusions

In summary, black TiOx films with high photothermal and photocatalytic activities were successfully fabricated by regulating the oxygen flow and target voltage in the reactive DC magnetron sputtering process. As the oxygen flow increased, the main phase changed from Ti to TiO, to Ti_2_O_3_, to Ti_3_O_5_, and TiO_2_. Among these oxides, Ti_2_O_3_ and Ti_3_O_5_ played a role in light absorbance, and TiO_2_ played the role of a semiconductor photocatalyst that formed an LASC system to improve the photocatalytic activity. By controlling the oxygen flow at 5.6 ± 0.2 sccm and target voltage near 400 V, the black TiOx films with the most Ti_2_O_3_ and crystallized Ti_3_O_5_ phases (Sample S3) could be fabricated. Sample S3 exhibited the highest absorbance and roughest surface in the samples, which led to the best photothermal effect and photocatalytic activity. The average optical absorbance of the films (Sample S3) reached up to 89%, and the surface temperature under sunlight could reach up to 114 °C. The photogenerated current could be increased by 15 times compared with TiO_2_ films; Rhodamine-B degraded to 74% after 150 min of UV irradiation. In conclusion, it is believed that the black TiOx films served as a photothermal and photocatalytic substrate that can pave a new path for designing more co-photocatalysts to achieve better efficiency.

## Figures and Tables

**Table 1:** The sputtering parameters and the average optical absorbance of Samples S1, S2, S3, and S4 from 250 to 800 nm.

Sample	Voltage/V	O_2_ Flow/sccm	Ar Flow/sccm	Average Absorbance/%
S1	360 ± 5	3.4 ± 0.2	36.6 ± 0.2	67.37
S2	380 ± 5	5.2 ± 0.2	34.8 ± 0.2	81.50
S3	400 ± 5	5.6 ± 0.2	34.4 ± 0.2	89.29
S4	420 ± 5	6.0 ± 0.2	34.0 ± 0.2	17.87

**Table 2:** O: Ti atomic ratio of the three samples and valence distribution of Ti element.

Sample	S1	S2	S3
O: Ti (XPS)	1.30	1.20	1.29
O: Ti (EDS)	0.67	1.07	1.23
Ti^4+^/%	30.73	43.89	44.27
Ti^3+^/%	19.65	24.41	26.44
Ti^2+^/%	39.01	31.70	29.29
Ti/%	10.61	0	0

**Table 3:** Roughness, thickness, and grain size of the films.

Sample	S1	S2	S3
PV/nm	29.90	49.98	58.76
RMS/nm	3.53	6.40	7.84
Ra/nm	2.79	5.08	6.26
Thickness/nm	580	700	800
Average particle size/nm	30.0	38.7	40.2

**Table 4:** The photocatalytic efficiency of some TiO_2_ films by sputtering methods.

Materials and Methods	Simulated Pollutant	Light Source	Degradation Rate/%·h^−1^
RF magnetron sputtering, TiO_2_ [20]	Rhodamine-B (0.5 mg/L)	8 W-254 nm	25
Pulse magnetron sputtering, TiO_2_ [21]	Methyl orange (10 mg/L)	36 W-365 nm	23
DC reactive sputtering ZnO_2_/TiO_2_ [33]	Methylene blue (10 mg/L)	15 W-254 nm	15–21
Glancing angle deposition [34], H-TiO_x_	Methylene blue (10 mg/L)	AM 1.5 G	22–31
RF magnetron sputtering, TiN/TiO_2_ [35]	Rhodamine-B (5 mg/L)	500 W full wavelength	20–40
Our work	Rhodamine-B (10 mg/L)	25 W-254 nm	13–30

## Data Availability

Data sharing not applicable.

## Supplementary Materials

The following are available online at https://www.mdpi.com/article/10.3390/ma14102508/s1. Figure S1: The binding energy of O 1s on the surface of the films before etching: (a) S1, (b) S2, (c) S3; the binding energy of O 1s on the surface of the films after etching: (d) S1, (e) S2, (f) S3; Figure S2: Selected area and fitting curve of Sample S1; Figure S3: Selected area and fitting curve of Sample S2; Figure S4: Selected area and fitting curve of Sample S3; Figure S5: The AFM images of these films: (a) S1; (b) S2; (c) S3; Figure S6: The cross-section SEM images of these films: (a) S1; (b) S2; (c) S3; Table S1: O 1s and Ti 2p binding energy and related fitting curves of sample S1; Table S2: O 1s and Ti 2p binding energy and related fitting curves of etched sample S1; Table S3: O 1s and Ti 2p binding energy and related fitting curves of sample S2; Table S4: O 1s and Ti 2p binding energy and related fitting curves of etched sample S2; Table S5: O 1s and Ti 2p binding energy and related fitting curves of sample S3; Table S6: O 1s and Ti 2p binding energy and related fitting curves of etched sample S3; Table S7: Element concentration of sample S1; Table S8: Element concentration of sample S2; Table S9: Element concentration of sample S3.
