# Supplementary material for: Black TiOx Films with Photothermal-Assisted Photocatalytic Activity Prepared by Reactive Sputtering
Source: Materials (Basel). 2021 May 12;14(10):2508. doi: 10.3390/ma14102508 (PMC8151715; doi:10.3390/ma14102508)
Supplement: Supplementary file 1 [file materials-14-02508-s001.zip › materials-1197277-supplementary.pdf]

materials

MDPI

Article

Black TiO_x Films with Photothermal-Assisted Photocatalytic Activity Prepared by Reactive Sputtering

Quan Mao [1,2], Meng Liu [1], Yajie Li [1], Yuquan Wei [1], Yong Yang [1,2,*] and Zhengren Huang [1,2,*]

[1] State Key Laboratory of High Performance Ceramics and Superfine Microstructures, Shanghai Institute of Ceramics, Chinese Academy of Sciences, 1295 Dingxi Road, Shanghai 200050, China; maoquan@mail.ustc.edu.cn (Q.M.); liumeng1@shanghaitech.edu.cn (M.L.); liyj@shanghaitech.edu.cn (Y.L.); weiyuquan@mail.sic.ac.cn (Y.W.)

[2] Center of Materials Science and Optoelectronics Engineering, University of Chinese Academy of Sciences, Beijing 100049, China

* Correspondence: yangyong@mail.sic.ac.cn (Y.Y.), Tel.: +86-21-69906065 (Y.Y.); zhrhuang@mail.sic.ac.cn (Z.H.), Tel.: +86-21-69906061 (Z.H.)

1. O1s XPS and Relative Fitting Curves Data

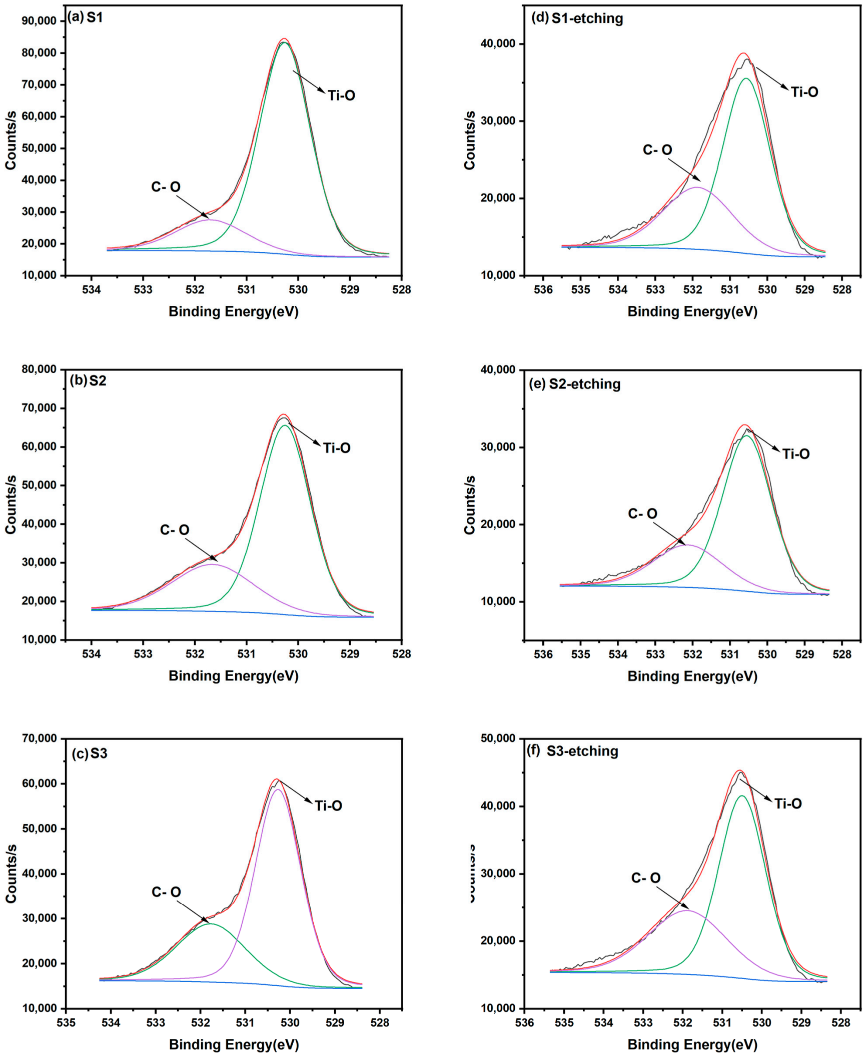

Figure S1. The binding energy of O 1s on the surface of the films before etching: (**a**) S1, (**b**) S2, (**c**) S3; the binding energy of O 1s on the surface of the films after etching: (**d**) S1, (**e**) S2, (**f**) S3.

Table S1. O 1s and Ti 2p binding energy and related fitting curves of sample S1.

Original S1	Binding Energy / eV	Full Width at Half Maximum / eV	Peak Area
C-O	531.70	1.59	18,260
Ti-O	530.26	1.15	89,551
Ti^{4+} $2p_{1/2}$	464.42	2.06	33,390
Ti^{4+} $2p_{3/2}$	458.74	1.17	93,434
Ti $2p_{3/2}$	454.28	1.73	8,572

Table S2. O 1s and Ti 2p binding energy and related fitting curves of etched sample S1.

Etched S1	Binding Energy / eV	Full Width at Half Maximum / eV	Peak Area
C-O	531.87	2.20	20,590
Ti-O	530.56	1.49	39,297
Ti^{4+} $2p_{1/2}$	463.64	2.84	9,571
Ti $2p_{1/2}$	460.46	2.25	21,955
Ti^{4+} $2p_{3/2}$	458.36	2.11	21,707
Ti^{3+} $2p_{3/2}$	456.44	2.11	19,209
Ti^{2+} $2p_{3/2}$	454.90	1.41	18,272
Ti $2p_{3/2}$	454.05	0.97	11,612

Table S3. O 1s and Ti 2p binding energy and related fitting curves of sample S2.

Original S2	Binding Energy / eV	Full Width at Half Maximum / eV	Peak Area
C-O	531.66	1.86	26,485
Ti-O	530.26	1.15	65,844
Ti^{4+} $2p_{1/2}$	464.42	2.49	32,098
Ti^{4+} $2p_{3/2}$	458.72	1.64	71,991
Ti^{2+} $2p_{3/2}$	455.73	2.61	8,572

Table S4. O 1s and Ti 2p binding energy and related fitting curves of etched sample S2.

Etched S2	Binding Energy / eV	Full Width at Half Maximum / eV	Peak Area
C-O	532.13	2.17	13,922
Ti-O	530.55	1.54	36,305
Ti^{4+} $2p_{1/2}$	463.56	3.16	14,077
Ti^{2+} $2p_{1/2}$	460.86	1.77	13,752
Ti^{4+} $2p_{3/2}$	458.46	2.19	28,019
Ti^{3+} $2p_{3/2}$	456.21	2.14	23,414
Ti^{2+} $2p_{3/2}$	454.77	1.19	16,659

Table S5. O 1s and Ti 2p binding energy and related fitting curves of sample S3.

Original S3	Binding Energy / eV	Full Width at Half Maximum / eV	Peak Area
C-O	531.77	1.81	27,265
Ti-O	530.27	1.20	61,107
Ti^{4+} $2p_{1/2}$	464.14	3.03	34,076
Ti^{4+} $2p_{3/2}$	458.77	1.52	60,248
Ti^{2+} $2p_{3/2}$	456.25	2.86	25,295

Table S6. O 1s and Ti 2p binding energy and related fitting curves of etched sample S3.

Etched S3	Binding Energy / eV	Full Width at Half Maximum / eV	Peak Area
C-O	531.88	2.33	25,648
Ti-O	530.50	1.40	44,230

Ti^{4+} $2p_{1/2}$	463.81	2.80	16,724
Ti^{2+} $2p_{1/2}$	461.01	1.89	16,887
Ti^{4+} $2p_{3/2}$	458.62	1.95	35,536
Ti^{3+} $2p_{3/2}$	456.42	2.16	31,215
Ti^{2+} $2p_{3/2}$	454.92	1.15	17,881

2. EDS Curves and Elements Composition of Samples.

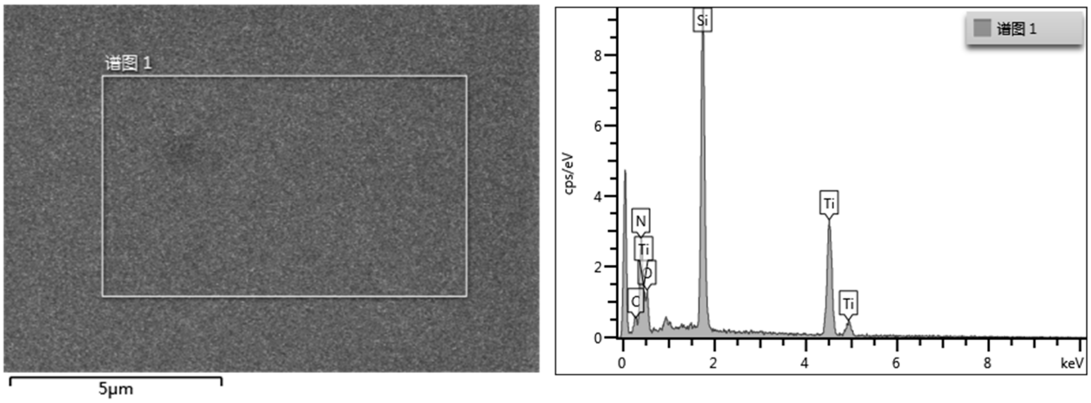

Figure S2. Selected area and fitting curve of Sample S1.

Table S7. Element concentration of sample S1.

Element	wt.%	wt.% Sigma	Atoms Ratio
C	4.12	0.55	9.43
N	8.91	1.08	17.46
O	11.20	0.82	19.23
Si	25.81	0.56	25.24
Ti	49.96	0.96	28.64
total:	100.00	-	100.00

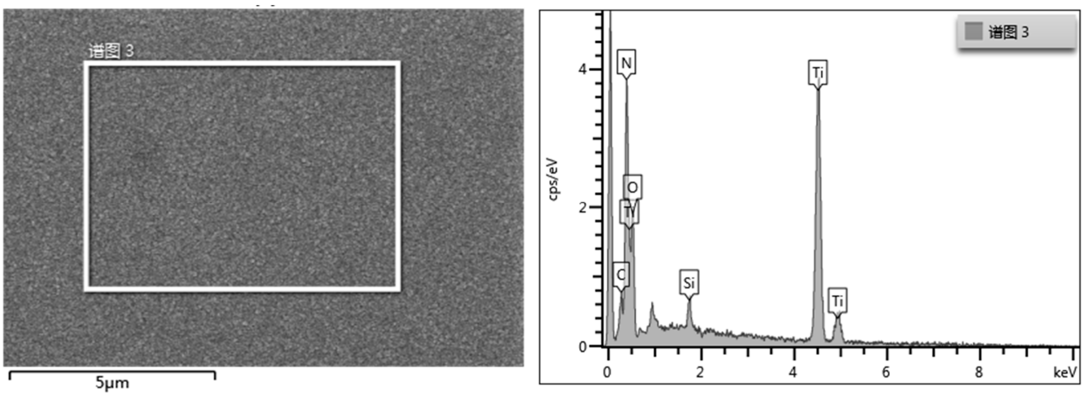

Figure S3. Selected area and fitting curve of Sample S2.

Table S8. Element concentration of sample S2.

Element	wt.%	wt.% Sigma	atoms Ratio
C	2.83	0.35	6.24
N	11.30	0.86	21.33
O	22.29	0.99	36.82
Si	1.39	0.15	1.31
Ti	62.18	1.07	34.31
total:	100.00	-	100.00

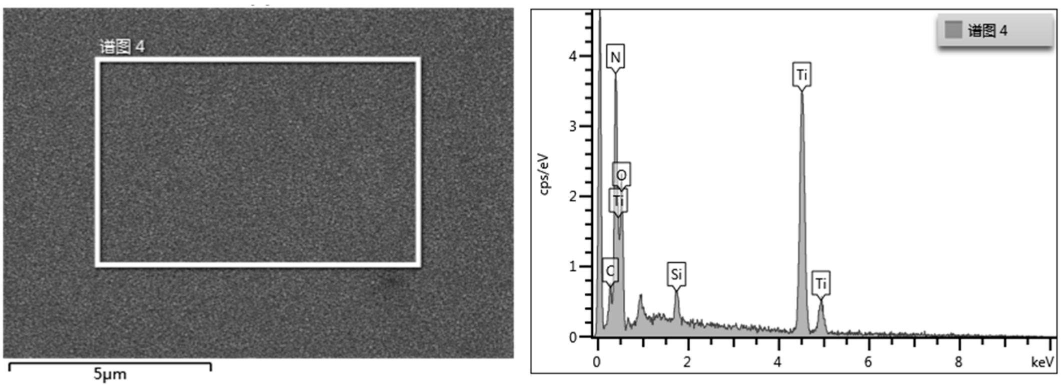

Figure S4. Selected area and fitting curve of Sample S3.

Table S9. Element concentration of sample S3.

Element	wt.%	wt.% Sigma	Atoms Ratio
C	3.02	0.35	6.45
N	11.55	0.87	21.15
O	24.48	0.99	39.24
Si	1.41	0.15	1.29
Ti	59.53	1.05	31.87
total:	100.00		100.00

3. AFM images

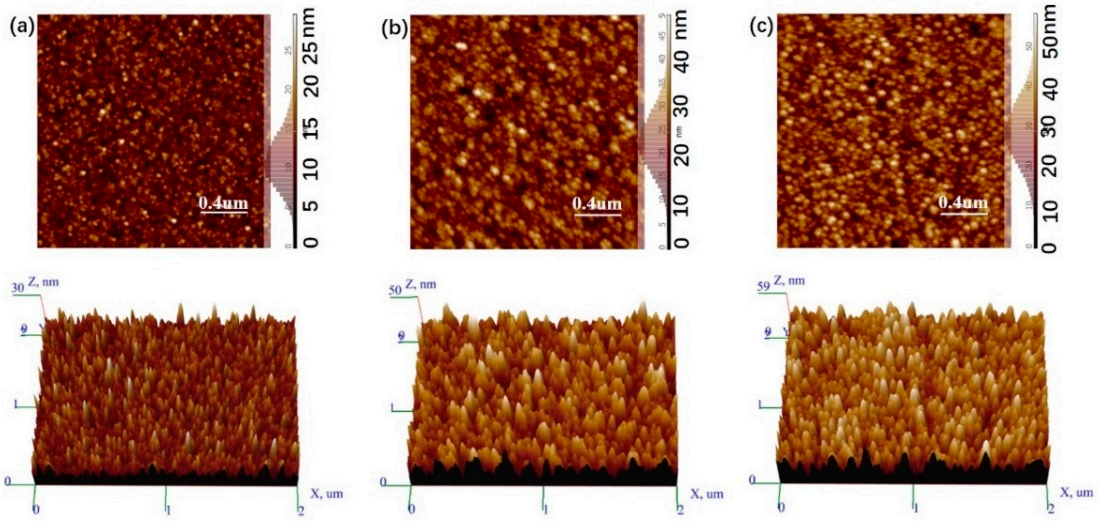

Figure S5. The AFM images of these films: (**a**) S1; (**b**) S2; (**c**) S3.

4. Cross-Section Morphologies of the Films

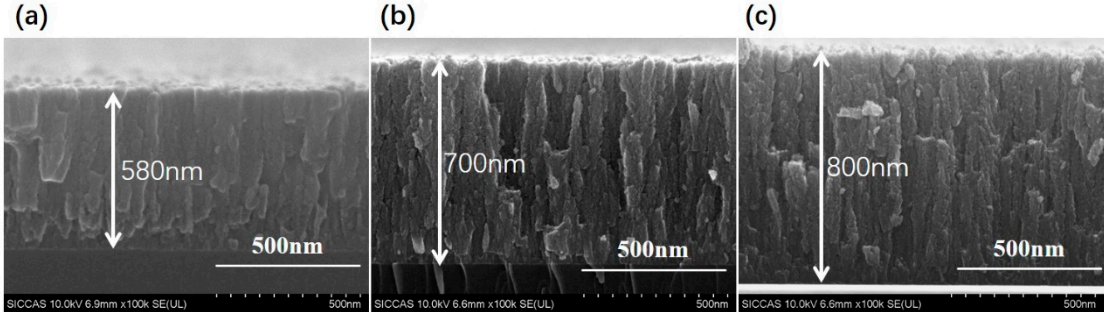

Figure S6. The cross-section SEM images of these films: (**a**) S1; (**b**) S2; (**c**) S3.
